# DAAM1 Is a Formin Required for Centrosome Re-Orientation during Cell Migration

**DOI:** 10.1371/journal.pone.0013064

**Published:** 2010-09-29

**Authors:** Su-Fen Ang, Zhuo-shen Zhao, Louis Lim, Ed Manser

**Affiliations:** 1 small G-protein Signaling and Kinases-Neuroscience Research Partnership (sGSK-NRP) Group, Agency for Science, Technology and Research (A*STAR), Singapore, Singapore; 2 Institute of Neurology, University College London, London, United Kingdom; 3 Rho GTPases in Stem Cells (RGS) Group, Institute of Medical Biology Agency for Science, Technology and Research (A*STAR), Singapore, Singapore; University of Washington, United States of America

## Abstract

**Background:**

Disheveled-associated activator of morphogenesis 1 (DAAM1) is a formin acting downstream of Wnt signaling that is important for planar cell polarity. It has been shown to promote proper cell polarization during embryonic development in both *Xenopus* and *Drosophila*. Importantly, DAAM1 binds to Disheveled (Dvl) and thus functions downstream of the Frizzled receptors. Little is known of how DAAM1 is localized and functions in mammalian cells. We investigate here how DAAM1 affects migration and polarization of cultured cells and conclude that it plays a key role in centrosome polarity.

**Methodology/Principal Findings:**

Using a specific antibody to DAAM1, we find that the protein localizes to the acto-myosin system and co-localizes with ventral myosin IIB-containing actin stress fibers. These fibers are particularly evident in the sub-nuclear region. An N-terminal region of DAAM1 is responsible for this targeting and the DAAM1(1-440) protein can interact with myosin IIB fibers independently of either F-actin or RhoA binding. We also demonstrate that DAAM1 depletion inhibits Golgi reorientation in wound healing assays. Wound-edge cells exhibit multiple protrusions characteristic of unpolarized cell migration. Finally, in U2OS cells lines stably expressing DAAM1, we observe an enhanced myosin IIB stress fiber network which opposes cell migration.

**Conclusions/Significance:**

This work highlights the importance of DAAM1 in processes underlying cell polarity and suggests that it acts in part by affecting the function of acto-myosin IIB system. It also emphasizes the importance of the N-terminal half of DAAM1. DAAM1 depletion strongly blocks centrosomal re-polarization, supporting the concept that DAAM1 signaling cooperates with the established Cdc42 associated polarity complex. These findings are also consistent with the observation that ablation of myosin IIB but not myosin IIA results in polarity defects downstream of Wnt signaling. The structure-function analysis of DAAM1 in cultured cells parallels more complex morphological events in the developing embryo.

## Introduction

Formins are an important class of actin nucleators. To date, 15 formin genes have been identified in mammals [1]–[3]. In metazoans, formins are classified into 7 subfamilies, termed Diaphanous (Dia), Disheveled-associated activator of morphogenesis (DAAM), formin related gene in leukocytes (FRL), formin homology domain containing protein (FHOD), formin-like protein (FMN), inverted formin (INF) and Delphilin. A typical formin contains a GTPase binding domain (GBD), formin homology (FH) 1, 2, 3 domains and a Diaphanous auto-regulatory domain (DAD). The GBD-DAD interaction maintains the formin in an auto-inhibited conformation [4]–[7]. Binding of an active Rho GTPase to the GBD relieves the auto-inhibition and activates the formin. Formins promote actin nucleation via the FH1 and FH2 domains [8]–[12], where FH1 is a proline-rich region that recruits profilin/actin. In recent years, formins like mDia1 have also been shown to regulate the microtubule network [13], [14].

DAAM1 was identified as an interactor of the PDZ domain of mouse Disheveled 2 (Dvl2) although not via conventional C-terminal sequences [15]; the mechanism by which Wnt-Dvl2 couples to downstream RhoA and Cdc42 stimulation is unclear, but likely involves one or several Rho-GEFs. In mouse, DAAM1 and DAAM2 are expressed in complementary patterns during mouse development consistent with tissues that require Wnt signaling. DAAM1 has been shown to be needed for *Xenopus* gastrulation; simultaneous loss of profilin and DAAM1 produce a synergistic inhibition of blastopore closure in *Xenopus* [16]. In *Drosophila*, DAAM1 plays an important role in the regulation of tracheal cuticle patterning via its effects on actin organization [17]. DAAM1 has been reported to interact with the Cdc42 effector protein CIP4 [18] which is involved in Arp2/3 mediated endocytosis. The crystal structure of DAAM1 FH2 domain [19], [20] reveals that the FH2 domain is oriented such that the actin binding surfaces are partially occluded. Thus a DAAM1 C-terminal fragment is a poor activator of actin polymerization *in- vitro* compared with the equivalent mDia1 fragment and suggests additional modes of regulation. Given the clear role of DAAM1 in the polarity of embryonic development, we have sought to further characterize DAAM1 with particular respect to its potential role in polarity in cultured mammalian cells. We describe targeting signals in the N-terminal of DAAM1 (distinct from the Dvl2 binding domain) that allows for targeted protein function. Significantly depletion of DAAM1 blocks normal centrosome/Golgi reorientation, showing that the protein cooperates in conventional cell polarization.

## Results

### DAAM1 localizes to acto-myosin stress fibers

**Figure 1:**
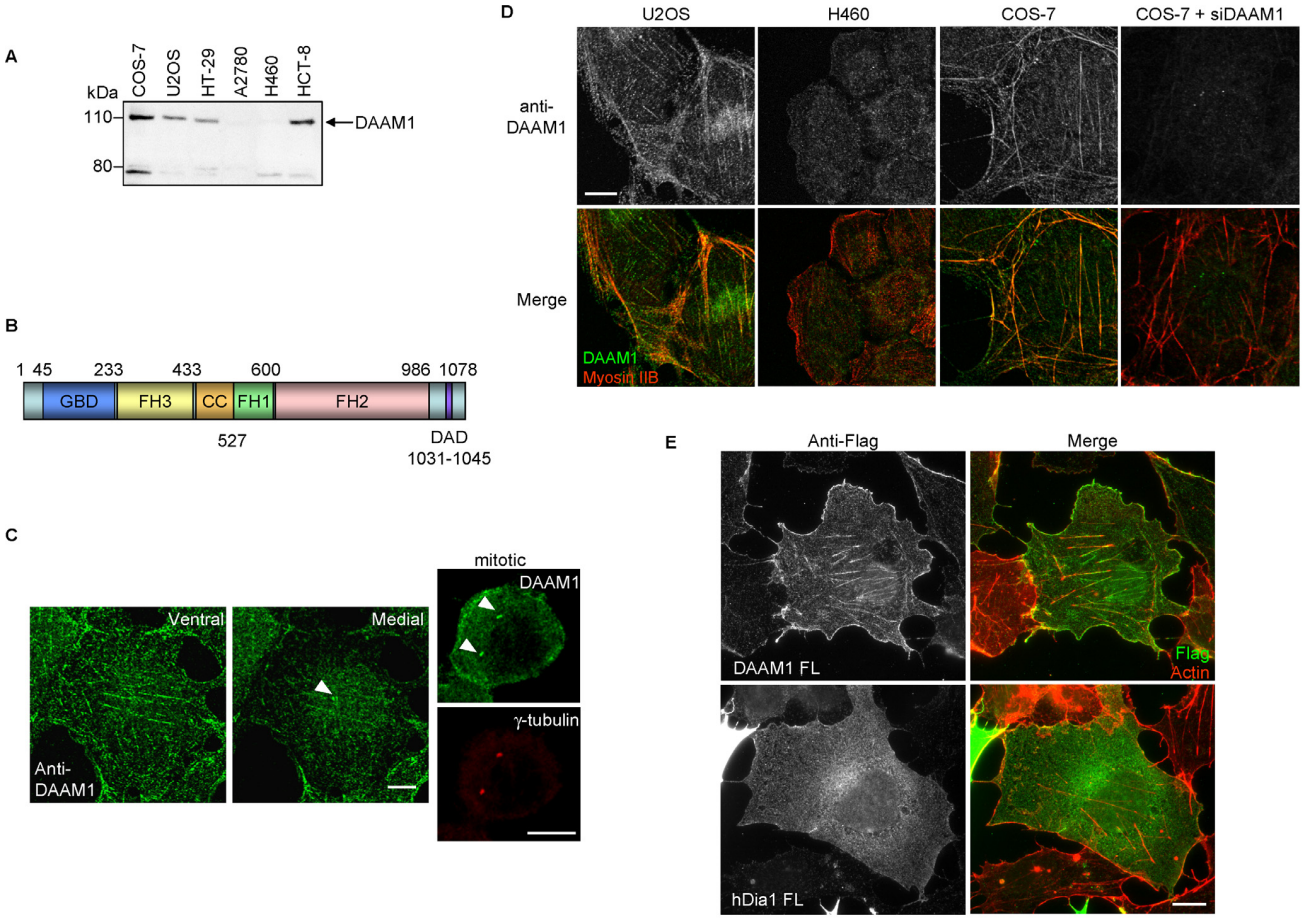
DAAM1 is localized to different regions in the cells. (A) Western analysis of DAAM1 expression in different cell lines. Total protein lysate (30 µg) were loaded per lane. (B) Domain organization of DAAM1. (C) Left: confocal images of COS-7 cell taken at different planes. Right: co-localization of DAAM1 with centrosomes in prophase (after centrosome duplication) marked by anti-γ-tubulin. White arrowheads indicate centrosomes. (D) Confocal images of U2OS and DAAM1-null H460 cells, as well as COS-7 and COS-7 cells with DAAM1 knockdown by siRNA (COS-7 + siDAAM1). Endogenous DAAM1 was detected by indirect immuno-fluorescence with anti-DAAM1 under identical conditions. (E) Full-length Flag-DAAM1 or Flag-hDia1 (in green) was expressed in COS-7 cells and co-stained for actin (red). Bars  = 10 µm.

We generated a polyclonal antibody to human DAAM1 (residues 596–1078), and used it to probe endogenous DAAM1 levels in a number of cell lines. By western analysis (Fig. 1A), we found significant heterogeneity of expression, with the 120 kDa protein absent in H460 and A2780 epithelial carcinoma lines. The nature of the smaller band is not known as small alternate transcripts of DAAM1 are not reported. DAAM1 is a formin with regions of sequence homology designated: GBD, FH3, FH1, FH2 and DAD as annotated in Fig. 1B, but nothing is known with respect to what domains are responsible for protein localization. In positive cell lines, endogenous DAAM1 showed evident co-localization with actin stress fibers particularly in the sub-nuclear region (Fig. 1C, ventral section), and on centrosomes (white arrowhead in Fig. 1C medial section). In mitotic cells which have enlarged centrosomes, confocal images showed clear staining at the spindle poles and in cortical regions adjacent to the plasma membrane (Fig. 1C, right panels). This colocalization with actin stress fibers by the anti-DAAM1 staining was confirmed by confocal imaging of DAAM1 positive COS-7 and U2OS lines and was absent from H460 lung cancer cells that lack DAAM1 (Fig. 1D). The stress fiber staining was abolished by siRNA treatment of COS-7 cells. A N-terminal tagged DAAM1 also associated with stress fibers, but not Diaphanous 1 (hDia1) that had a more diffused localization (Fig.1E).

### The DAAM1 N-terminal region binds to myosin IIB containing stress fibers

**Figure 2:**
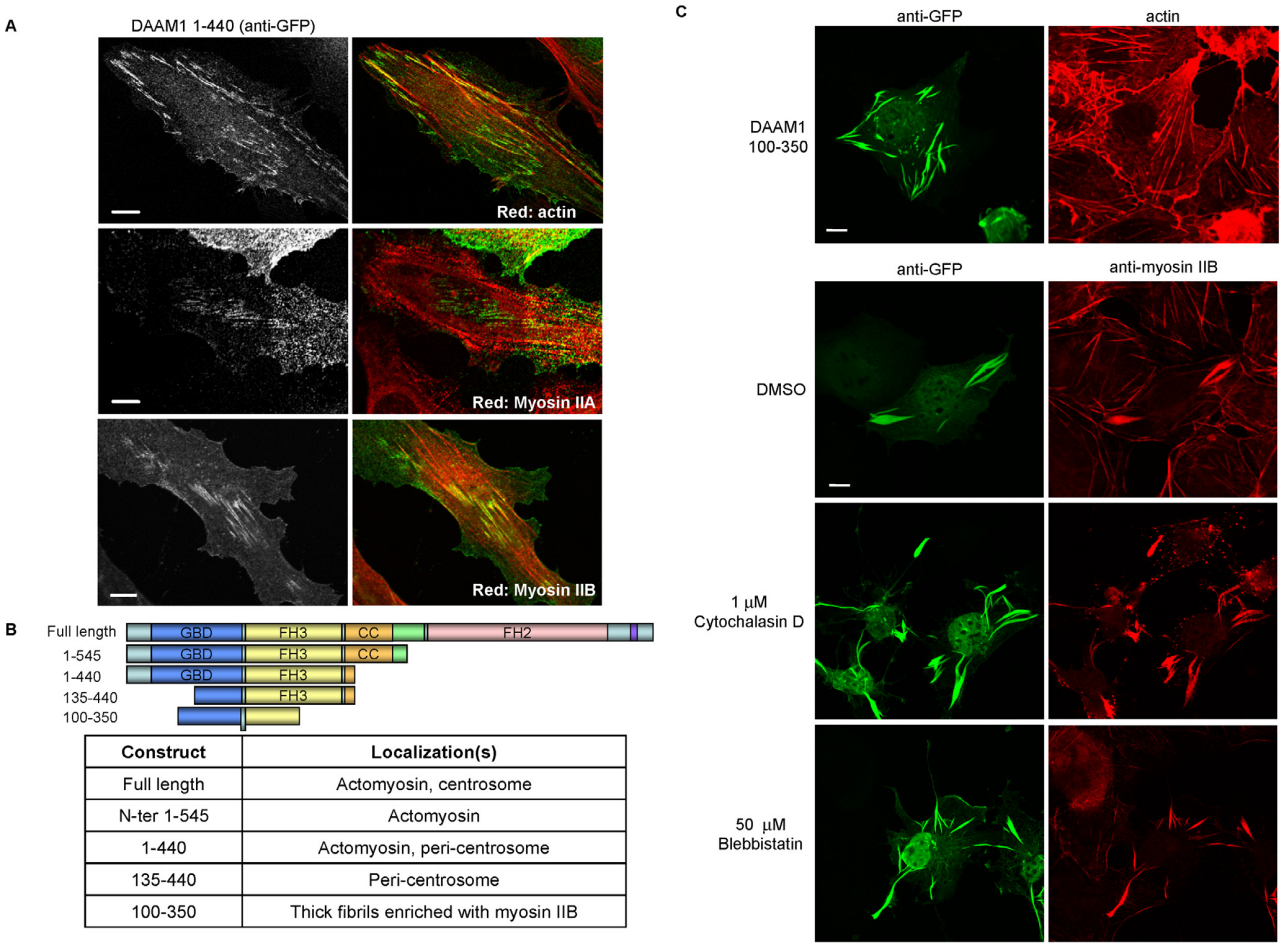
N-terminal regions of DAAM1 are involved with actin stress fibers and centrosome targeting. (A) GFP-Flag-DAAM1 (1–440) was expressed in HeLa and analyzed by confocal imaging. DAAM1 was immuno-localized with anti-GFP (in green). Cells were counter-stained with TRITC-phalloidin, anti-myosin IIA or anti-myosin IIB (red). (B) Table summarizing the localization of various DAAM1 constructs tested in this study. (C) COS-7 cells expressing DAAM1(100–350) form large filaments enriched for myosin IIB that do not colocalize with F-actin. Treatment with various acto-myosin disrupting drugs with the indicated concentration for 45 min do not abolish the presence of these fibrils. Bars  = 10 µm.

It is reported that the ‘membrane’ localization of mDia1 requires the N-terminal half and is negatively controlled by auto-inhibitory contacts [21]. In our initial experiments, it was clear that DAAM1-N(1–545) was localized similarly to full-length DAAM1, the same applies to the shorter DAAM1 (1–440) that retains the FH3 domain encompassing 235–433 [22]. In HeLa cells, DAAM1(1–440) localized more clearly to puncta along actin-myosin II stress fibers, and only a subset of actin fibers co-stained with DAAM1 (Fig. 2A, panel 1). The fiber staining showed no significant co-localization with myosin IIA (panel 2) but to myosin IIB (panel 3). Localization of DAAM1(1-440) to the actin-myosin II stress fibers was abolished by the deletion of the first 134 residues. DAAM1(135–440) instead localized only to the peri-centrosomal area (unpublished data). A summary of the DAAM1 regions contributing to protein localization is shown in Fig. 2B. It is notable that the Dvl2-binding domain is located in the FH2-DAD region [19] and thus not implicated in this DAAM1 localization. Remarkably a construct encompassing residues 100–350 (including a region of the first putative coiled-coil) generated thick DAAM1 fibrils that recruited high levels of endogenous myosin IIB, (Fig. 2C). This interaction between myosin IIB and DAAM1(100–350) did not require either F-actin nor myosin contractility as assessed by treatment of the cells with specific inhibitors (Fig. 2C). In DAAM1(100–350) expressing cells, the majority of myosin IIB became associated with DAAM1: to our knowledge there is no abundant myosin II binding protein that might mediate this interaction. Biochemical analysis of this interaction was not possible due to the detergent insoluble nature of DAAM1(100–350).

### DAAM1 localization to actin stress fibers is independent of Rho GTPase binding

**Figure 3:**
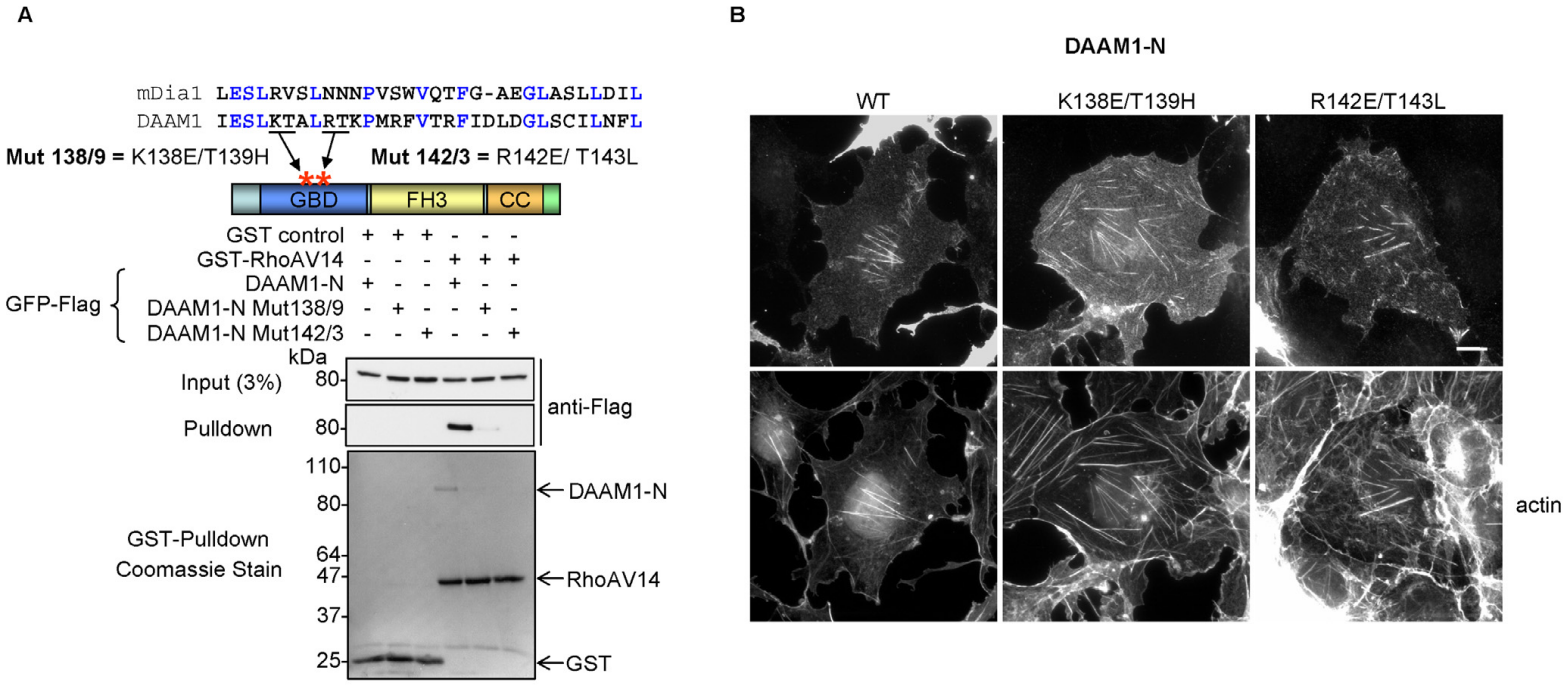
Interaction of DAAM1 with Rho GTPases. (A) The design of putative Rho binding defective mutants was based on sequences in mDia1: RhoA complex [6]. An *in-vitro* pulldown assay was performed in which equal amounts of DAAM1-N wildtype (WT) or the GBD mutants were added to Sepharose beads loaded with GST or GST-RhoAV14. The proteins were transferred to PVDF and stained as shown. The membrane was subsequently probed as indicated (B) GFP-DAAM1-N WT or the GBD mutants were expressed in COS-7 cells and immuno-localized with anti-GFP and F-actin (TRITC-phalloidin); all three constructs show typical DAAM1 filamentous staining. Bars  = 10 µm.

Since the region of DAAM1 involved with stress fiber localization overlap with the GTPase-binding domain (GBD), we were interested to test if Rho interaction was necessary. We first tested the ability of three ubiquitous Rho GTPases (RhoA, Rac1 and Cdc42) to bind DAAM1 *in vivo*. This region (DAAM1-N) bound all three GTPases (Fig. S1) with essentially equal efficiency (i.e. comparing input and pull-down bands). The GBD of formins like Bni1p and mDia1 [23], [24] can be aligned to that of DAAM1. We generated two GBD mutants, K138E/T139H and R142E/T143L, based on analogous residues on the protein surface that play a role in RhoA binding to mDia1 [4], [5], [7]. These DAAM1 GBD mutants showed severely reduced RhoA.GTP association in an *in-vitro* binding assay (Fig. 3A) and in cotransfections. There was no difference in the stress fiber localization between GFP-DAAM1-N and corresponding K138E/T139H or R142E/T143L mutants (Fig. 3B). Thus DAAM1 does not require RhoA binding to associate with the acto-myosin network. With respect to assessing specifically which endogenous Rho proteins activate DAAM1 *in-vivo*, conformationally sensitive antibodies that detect this state would be needed. We conclude Rho GTPases primarily play a permissive role with respect to FH2 activity of DAAM1 and do not significantly affect the localization of the protein. Recently Ju et al. reported that DAAM1 has specific roles in endothelial cell proliferation via selective effects on microtubules [25]. Since these effects were based on the expression of the FH1/FH2 C-terminal half (i.e. without appropriate targeting sequences) it is unclear if such experiments are informative.

### DAAM1 knockdown severely impairs centrosome re-orientation in monolayer scratch assays

RhoA and DAAM1 are needed to establish planar cell polarity during early development in vertebrates and invertebrates [15], [16]. Centrosomal polarization by contrast requires a Cdc42-dependent pathway involving the PAR3/PAR6/aPKC complex rather than RhoA signaling [26]. In scrape-wound assays of cell monolayers, wound-edge cells orient their centrosomes towards the wound, a process that is easy to monitor, and often represented as process driven by new extracellular-matrix adhesions recruiting polarization/PAR proteins to the leading edge. However, a recent study using cells grown on micro-patterns questions this notion since isolated cells without cell-cell contacts cannot polarize in response to adhesion [27]. Rather asymmetry (absence) of cell-cell adhesions drives cell polarization: disrupting E-cadherin engagement between cells abrogates scrape-wound-induced centrosomal reorientation, though not cell migration. Cell-cell adhesions can induce displacement of the nucleus towards these contacts, and away from the free edge [27]. This nuclear displacement as a result of wounding is known to require contractile myosin II driven by the Cdc42 effector MRCK [28], causing an illusion of “centrosomal reorientation”. The polarity protein PAR3 associates with dynein to maintain a central position of the centrosome at the cell center [29], and in wound-edge fibroblasts this PAR3 is localized to cell-cell contacts and not the leading edge.

**Figure 4:**
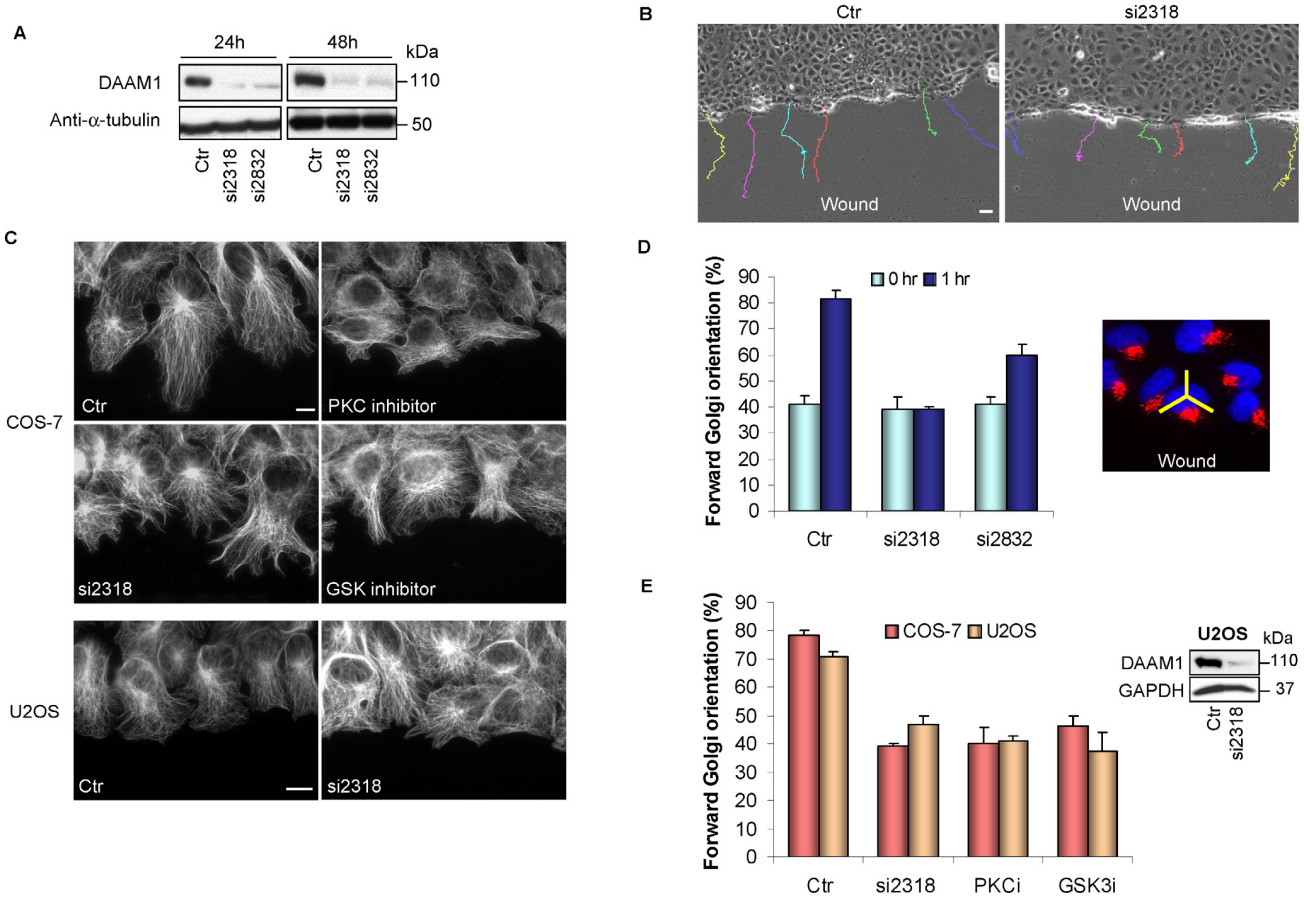
DAAM1 knockdown affects directed cell migration. (A) Efficacy of two different siRNA against DAAM1 in COS-7 cells by Western analysis at 24 hours and 48 hours post-transfection. (B) COS-7 cells transfected with control (Ctr) or human DAAM1 siRNA (si2318) for 48 hours were scratched and observed using time-lapse imaging for 12 hours. Tracks of individual cells are shown as colored lines. Bar  = 40 µm. (C) Wound-edge COS-7 cells subjected to GSK inhibitor (SB216763, 20 µM) and PKC inhibitor (RO-320432, 20 µM), or si2318 (40 pmol) were stained with anti-α-tubulin to visualize the microtubule network. Loss of DAAM1 was associated with random protrusions versus the more organized broad extensions in controls. Wound-edge U2OS cells subjected to siRNA treatment are shown in the bottom panel. Bar  = 10 µm. (D) Forward Golgi orientation of COS-7 cells transfected with control (Ctr) or DAAM1 siRNA (si2318 or si2832, 40 pmol) at 0 and 1 hour post-wounding. Cells were scored for Golgi re-orientation using a 120° sector centered on the nucleus as shown on the right. (E) Right: Western analysis of DAAM1 knockdown in U2OS cells. Left: Golgi re-orientation in COS-7 and U2OS cells treated with various inhibitors 1 hour before wounding. PKC inhibitor (PKCi) and GSK inhibitors (GSKi) were used as in (c). In these analyses, cells were scored for Golgi re-orientation in three separate experiments. Error bars indicate standard deviation from the mean.

That DAAM1 selectively associates with these sub-nuclear stress fibers suggests this might be a site of action. Recently there is evidence that Dvl and Axin, (i.e. components of the Wnt pathway) also play a role in centrosome reorientation in monolayer scratch assays [30]. To test if DAAM1 is involved in cell polarization, two siRNA pairs (si2318 and si2832) were used to knock down DAAM1 expression in COS-7 cells (Fig. 4A). After 48 hours, COS-7 cell monolayers were scratched and cell migration was monitored by time-lapse microscopy (Fig. 4B). In DAAM1 knockdown cells, we observed multiple branched protrusions with a less organized microtubule network, while control siRNA-treated cells in general showed a single broad lamella pointing into the wound (Fig. 4C). Further DAAM1 siRNA clearly caused cells to migrate in a random fashion (Movies S1 and S2), indicating a loss of directionality as seen by single cell tracing (colored lines, Fig. 4B) consistent with loss of other polarity components such as Par3 [29]. Rates of cell migration were not significantly different from controls indicating no perturbation to the Rac1 pathway. These profound defects in polarity due to DAAM1 knockdown were seen in both COS-7 and U2OS cells which are of quite different origins (Fig. 4D and 4E). An entirely random orientation of the Golgi (towards the forward 120° sector of the scratch) gives a 33% baseline. The blockage of cell orientation by DAAM1 depletion was as effective as inhibition of aPKC or GSK3b (Fig. 4E) which are kinases essential for polarization [31], [32].

### Elevated DAAM1 enhances myosin IIB stress fibers and impairs cell migration

**Figure 5:**
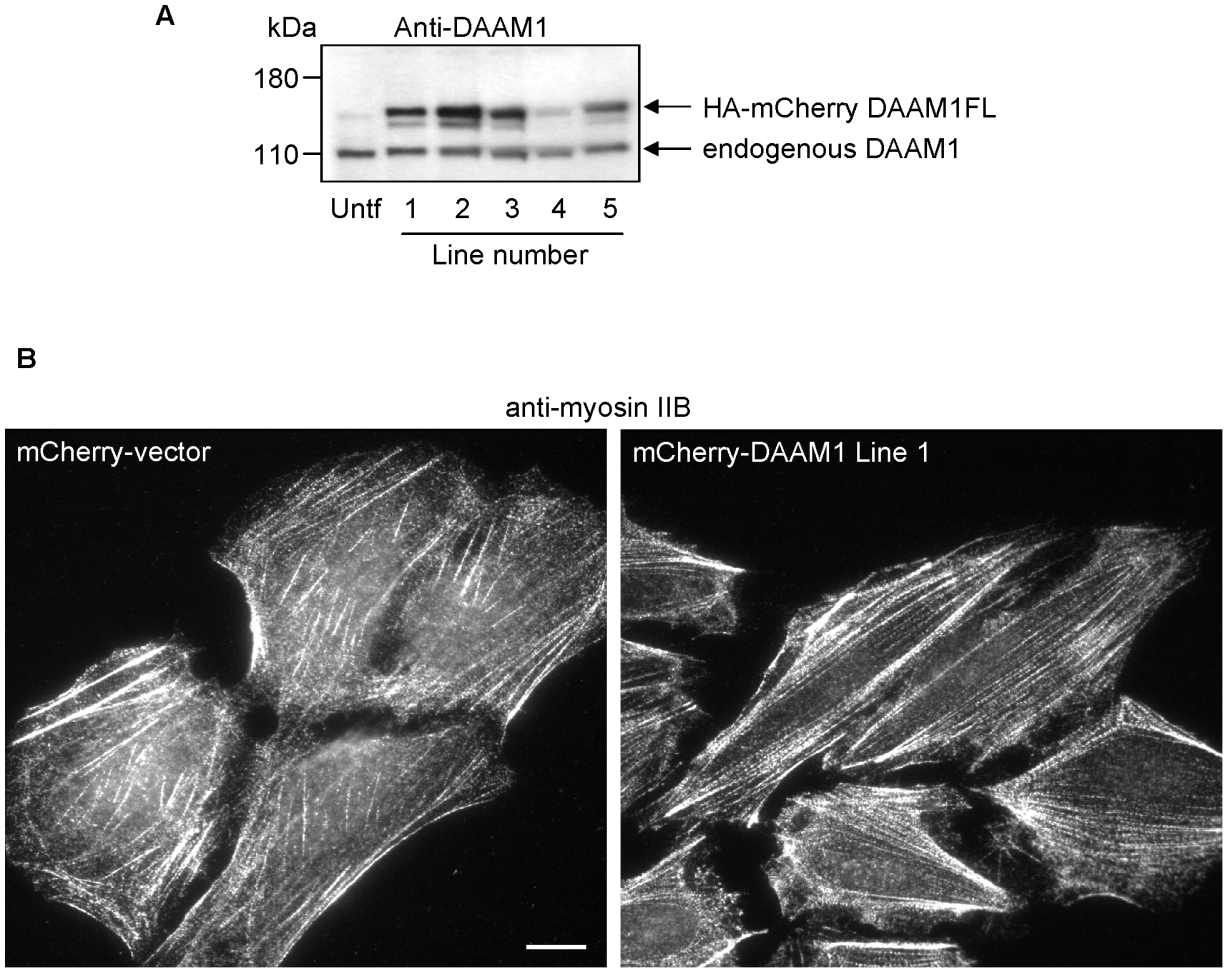
Stable expression of DAAM1 in U2OS cells. (A) Levels of mCherry-DAAM1 and endogenous DAAM1 in the five stable U2OS lines as determined by western analysis with anti-DAAM1. (B) Typical myosin IIB staining of the ventral membrane region for the control and mCherry-DAAM1 expressing cells. Bars  = 10 µm.

Having established that knockdown of DAAM1 profoundly affects polarization, we finally investigated the behavior of stable U2OS cell lines expressing mCherry-DAAM1. We selected five different lines expressing 2–4 times endogenous DAAM1 levels (Fig. 5A). We found that there was little difference in the organization of the myosin IIA versus parental U2OS cells (data not shown). By contrast, myosin IIB positive stress fibers were clearly more abundant and well organized in the mCherry-DAAM1 lines as compared to the controls (Fig. 5B).

**Figure 6:**
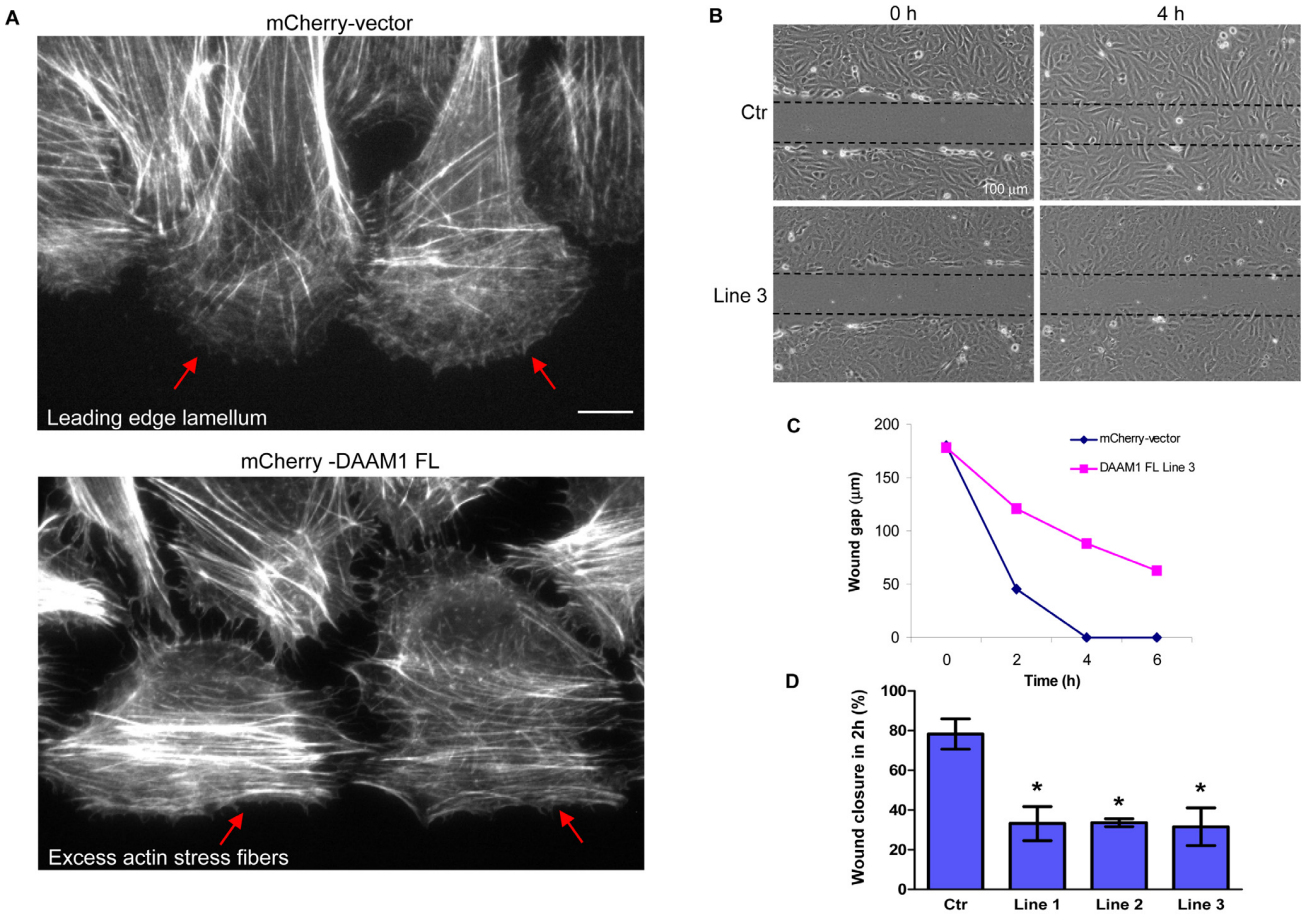
Effects of stable DAAM1 expression in U2OS cells. (A) Cells stably expressing mCherry alone or mCherry-DAAM1 as shown in Fig. 5 were subjected to wound healing assays and stained with Alexa Fluor 633-phalloidin to visualize the actin fibers. Red arrows indicate the different arrangement of actin fibers at the leading edges of vector control and DAAM1-expressing cells. Red arrows indicate the lamella region largely devoid of stress fibers in controls (top panel red arrows), and the presence of prominent actin fibers instead for the DAAM1-expressing cells (lower panel). (B) Cell lines stably expressing the mCherry vector or mCherry-DAAM1 were subjected to scratch wounding (wound indicated by dotted lines) and images were acquired at 2 hour intervals over 6 hours using an Olympus IX71 microscope equipped with a 10x/0.25 Plan-APOCHROMAT lens. Images at 0 and 4 hour time points are shown here for control and the DAAM1 cell-line 3. (C) Line graph showing the reduction in wound gap over the entire course of 6 hours for the control line and DAAM1-expressing line 3. (D) Percentage of wound gap covered 2 hours after scratch is plotted for control versus three of the lines. Error bars indicate standard error of the mean (SEM) from 3 independent experiments. Asterisks indicate that difference in the measurement between the control and each DAAM1 expressing line is statistically significant with a p-value less than 0.05 (t-test). Bars  = 10 µm (unless specified).

In standard monolayer scratch assays, cells expressing mCherry-DAAM1 differed from controls in terms of focal adhesion and actin stress fiber distribution (Fig. 6A). Images of typical leading edge cells of the mCherry-DAAM1 lines are shown in Fig. S2. Upon cell migration into the open area of the ‘wound’, focal adhesions are usually disassembled from the cell center and re-distributed towards the membrane extension at the cell edge. In the DAAM1 lines, cells exhibited focal adhesions that remained distributed throughout the cells, which is similar to those of non-wound edge cells. The actin stress fibers are normally re-organized to facilitate migration - which involves loss of RhoA-type myosin IIB [33] and re-organization of the MRCK-driven myosin IIA fibers [34]. Time-lapse imaging showed that U2OS cells migrate rapidly into the wound and were able to close the wound in approximately 4 hours. By contrast, all the mCherry-DAAM1 lines exhibited delayed cell migration which was clearly seen at the 4 hour time point (Fig. 6B). The line graph in Fig 6C depicts the migration distance for control and mCherry-DAAM1 line 3 at 2 hour intervals (see Fig. S3 for corresponding wound images). The average distance covered by leading edge cells (measured in terms of percentage of wound covered in 2 hours) is shown for control versus 3 DAAM1 expressing lines (Fig. 6D). Since myosin IIB is associated specifically with impaired migration downstream of RhoA [33], [35], the slower migration rates are consistent with the selective up-regulation of myosin IIB-containing actin stress fibers as a result of DAAM1 expression.

**Figure 7:**
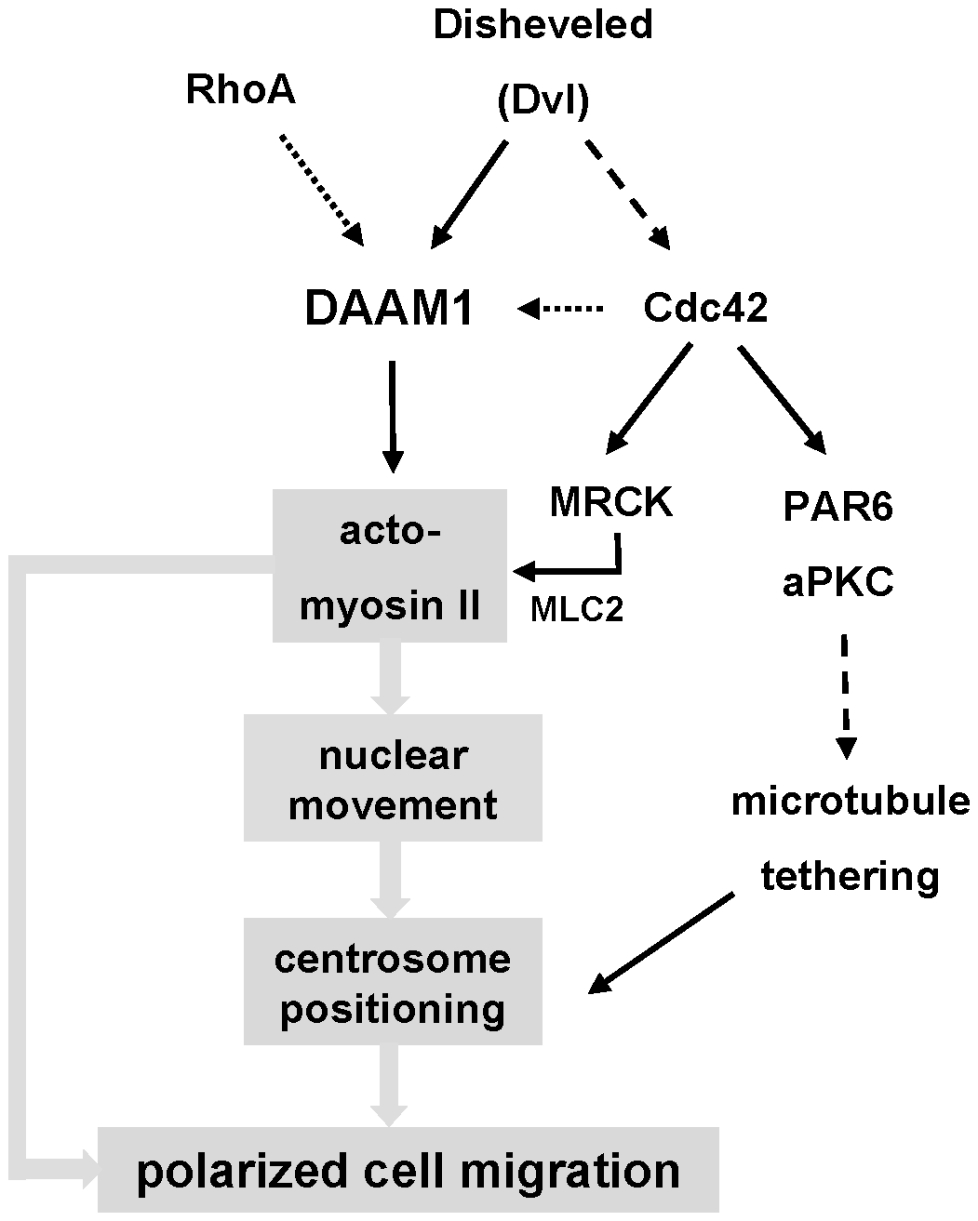
A model of DAAM1 involvement in directed cell migration. Through its effects on F-actin assembly and the propensity to associate with myosin IIB, DAAM1 can directly affect the regulation of the acto-myosin network. Its upstream activator, Disheveled, is involved with activation of other polarity proteins through Cdc42 [30]. Cdc42 and MRCK are implicated in nuclear movement via effects on the sub-nuclear acto-myosin network [28], [34]. PAR3/PAR6/aPKC and dynein maintain the centrosome in the cell centroid via their effects on microtubule organization [29]. The ability of DAAM1 to interact with RhoA and Cdc42 could be a focus of cooperativity between the two GTPases in the polarity pathway. Full arrows indicate direct interaction, dashed arrows indicate indirect interaction, and dotted arrows indicate interaction yet to be established in this pathway.

These experiments suggest that DAAM1 plays a role in the assembly of actin stress fibers as indicated in Fig. 7. In addition to binding Disheveled proteins [36], DAAM1 associates with certain myosin IIB complexes in the cell. Increased DAAM1 levels enhance the acto-myosin machinery which restricts cell movement. By contrast, loss of DAAM1 blocks centrosomal re-positioning during cell migration, resulting in a loss of directionality of the cell monolayer during cell migration into a ‘wound’. Whether DAAM1 exerts its effect on centrosome/nuclear positioning directly or indirectly through its effects on the actin stress fibers remains to be established. The role of the acto-myosin II system in repositioning the nucleus requires the Cdc42 effector MRCK [28]; it is notable that DAAM1 is enriched on the sub-nuclear stress fibers where MRCK is found [34].

## Discussion

### The DAAM1 N-terminus provides localization cues

We describe here for the first time a localization of the formin DAAM1 to a specific actin/myosin II compartment. This localization is stronger with the isolated N-terminal regions (1-440), consistent with auto-inhibition of the N-terminus [37] by the C-terminal DAD region. This is consistent with the notion that N-terminal regions of DAAM1 that provide these localization cues are distinct from the catalytic region of formins that promotes F-actin assembly [8]. Expression of the constitutively active FH1-FH2 fragment of DAAM1 induces inappropriate actin polymerization and microtubule stabilization, which inhibits endothelial cell growth [25]; the authors also note that the N-terminus appears to behave as a dominant inhibitory construct indicating it competes for binding of some cellular factor(s) as suggested by our findings. We show here that assembled myosin IIB is a target for DAAM1; this may occur at the interface between the Dvl-DAAM1 complex at the plasma membrane which would explain the selective staining of stress fibers. This reveals a new paradigm where myosin II fibrils can, through associated formins such as DAAM1, initiate nucleation of actin fibers that allow proper assembly of contractile acto-myosin units. The disposition of additional formins needs to be investigated. In the context of the mCherry-DAAM1 expressing U2OS cell lines (Fig. 5B), we have noted that modest increases in the level of this formin can promote myosin IIB-containing actin stress fibers. Controlled by various myosin light chain kinases [33], these filaments can antagonize cell migration by promoting attachment [35], [38]. These findings are also consistent with the observation that ablation of myosin IIB but not myosin IIA results in polarity defects downstream of Wnt signaling [39], [40].

### Interaction of DAAM1 with multiple Rho GTPases

DAAM1-N was found here to interact with three representative members of the Rho family. This is at variance with previous reports for its RhoA specificity [15], [18], but in agreement with an ability to complex with both Cdc42 and CIP4 [18]. We suggest that the GTPase partner of choice may be dependent on which Rho GTPases are activated in the vicinity of DAAM1 (Fig. 7). An ability of DAAM1 to bind Cdc42 as well as RhoA would support its key role in cell polarization events. We have explored the idea of mutating DAAM1 in order to selectively interfere with DAAM1 interaction with RhoA and not Cdc42, but have been unsuccessful to date. DAAM1 can localize to the centrosome/Golgi region where Cdc42 has been reported to act [41], whereas plasma membrane Dvl2-DAAM1 may be activated primarily by RhoA.GTP. The identity of the putative DAAM1 associated RhoA-GEF involved in Wnt signaling has not been established [15]. The involvement of DAAM1 at the centrosome links it to Cdc42, PAR6, and PKCζ as well as the Wnt pathway that promote cell polarity [30], [32]. DAAM1 has the right credentials for a role in mediating actin-centrosome events, as invoked for mDia in microtubule stability [14].

### DAAM1 is a formin that affects both cell polarity and movement

It is clear from our study that DAAM1 has discrete N-terminal functions that allow it to localize in cells. There is a tight link between the position of the Golgi and the centrosome in most cells. Wound-induced Golgi and centrosome reorientation occurs via conserved signal transduction pathway(s) [42]–[45]. The positioning of the Golgi apparatus reflects the centrosome position and promotes directional vesicular transport to facilitate cell movement [46]–[48]. It is clear that loss of DAAM1 (in two cell types) impairs Golgi orientation and leads to more random migration (Fig. 4). Golgi organization in U2OS cells was also perturbed by subtle increased levels of DAAM1, suggesting that an optimal level of DAAM1 is important for Golgi integrity. It is not certain if this is a direct effect of DAAM1 on the Golgi or an indirect effect of DAAM1 on the cytoskeletal structures (cf. microtubules, actin). The microtubule network was not different in the mCherry-DAAM1 stable lines which showed Golgi dispersion (unpublished data).

### Role of myosin IIB associated DAAM1 in cell migration

It has been reported that myosin IIB plays a specific role in establishing front-back polarity and centrosome orientation [38], [49]. Myosin IIB-null fibroblasts have multiple disorganized lamellipodia [49]. This is similar to the phenotype observed in wound edge COS-7 cells depleted of DAAM1: cells were capable of moving but the protrusions were random (Fig. 4). Conversely, overexpression of DAAM1 in U2OS cells result in a different organization of the focal adhesions and the actin stress fibers of the wound edge cells, indicating a loss of the ability to protrude normally (Fig. 6). It is interesting to note that myosin IIB (versus myosin IIA) is selectively required for convergent extension in *Xenopus* gastrulation [39], [40], downstream of the non-canonical Wnt-RhoA pathway. This regulation of contractile forces within the cortical actin network facilitates the intercalation of cells. Selective ablation of myosin IIB resulted in polarity defects which are manifested phenotypically as the inability of blastopore and neural tube closure [39], [40].

In conclusion, our new findings that DAAM1 is coupled directly to myosin IIB adds weight to the model that the formin can link F-actin assembly to myosin IIB assembly, in a signaling pathway involving polarized movement, like convergent extension during gastrulation or directional migration in cultured cells. The positioning of DAAM1 on myosin IIB is highly suggestive that myosin filaments can attract nucleators of actin filaments to produce functional acto-myosin assemblies (Fig. 7). These processes could then promote nuclear movement and centrosome positioning during cell polarization. Future detailed studies of the interaction of DAAM1 with protein partners via its N-terminal half should promote understanding of how and when the formin is recruited to the various cellular sites. An interplay between kinases such as MRCK [28], [34] and ROK [33] that regulate contractility, and formins such as DAAM1 is anticipated. It has been demonstrated that the formin FHOD3 is required for organization of the sarcomere (the acto-myosin unit) in cardiac myocytes [50]. Although we presently understand little of the dynamic assembly of the acto-myosin network in non-muscle cells, coordination between myosin II assembly and F-actin assembly is envisaged.

## Materials and Methods

### Cell culture

A2780 (ECACC no. 93112519), H460 (ATCC no. HTB-177), HCT-8 (ATCC no. CCL-244) and HT-29 (ATCC no. HTB-38) were cultured in RPMI media supplemented with 10% fetal bovine serum (FBS) and 2 mM L-glutamine. HeLa cells (ATCC no. CCL-2) were cultured in minimum essential medium (MEM) supplemented with 10% FBS, 2 mM L-glutamine, 10 mM sodium pyruvate, 0.15% w/v sodium bicarbonate, and 0.1 mM MEM nonessential amino acids (Invitrogen). COS-7 (ATCC no. CRL-1651) and U2OS (ATCC no. HTB-96) cells were cultured in Dulbecco's modified Eagle's medium (DMEM) supplemented with 10% FBS. Cells were grown in a 37°C incubator with 5% CO_2_.

### Antibodies

Rabbit polyclonal and mouse anti-FLAG antibodies were from Sigma (St. Louis, MO). Monoclonal anti-GM130 was from BD Transduction Labs (San Jose, CA). Monoclonal anti-α-tubulin, anti-γ-tubulin and rabbit polyclonal anti-gamma tubulin were from Sigma. Polyclonal goat anti-rabbit and rabbit anti-mouse IgG HRP were from Dako Cytomation (Glostrup, Denmark). Alexa Fluor 488, 546, 633 and 647 fluorescent secondary antibodies were from Molecular Probes, Invitrogen. Rabbit polyclonal antibody against human DAAM1 was raised against recombinant His-tagged DAAM1 (594–1,078) in rabbits (Genemed Synthesis Inc., South San Francisco, CA). The antibody was purified using cyanogen bromide coupled to DAAM1 (594–1078) and eluted at pH 2.5.

### Other reagents

GSK-3 inhibitor SB216763 was from Sigma. PKC inhibitor RO 32–0432 was from Calbiochem (Darmstadt, Germany). Geneticin was from Invitrogen. TRITC-phalloidin and DAPI was from Sigma. Alexa Fluor-tagged phalloidin were from Molecular Probes.

### Generation and site-directed mutagenesis of expression plasmids

Human DAAM1 full-length DNA (KIAA0666) was obtained from Kazusa DNA Research Institute (Chiba, Japan). Different DAAM1 truncation constructs were generated by PCR, cloned into pXJ40-GFP-FLAG vector (XhoI/KpnI) or Geneticin-resistant pcDNA-HA-mCherry vector (for generation of stable cell line). Constructs were completely sequenced. GST-tagged Rho GTPases were kindly provided by Dr Dong Jing Ming. Human Diaphanous 1 (hDia1) constructs were kindly provided by Dr Koh Cheng Gee. The DAAM1 N-terminal point mutations were performed using a two-step PCR method. The integrity of resulting mutations was confirmed by DNA sequencing.

### Transfection of siRNA

Cells were transfected with either 40 pmol or 80 pmol of DAAM1 siRNA using Lipofectamine (Invitrogen) and incubated for 48 hours before harvesting (for Western analysis) or fixation (immunofluorescence). The forward strands of the DAAM1 siRNA were si2318 = 5′-GGUUGCAAUCGCUGUACUU-3′ and si2832 = 5′-CCUUCUAGCAGAAGCUAAA-3′ corresponding to the nucleotide position. An unrelated control siRNA sequence used is 5′-GCUGUCACAGGGGAGUUUACG-3′.

### Immunoprecipitation

COS-7 cells seeded on 100 mm dishes were transfected at 80% confluency using Lipofectamine (Invitrogen), harvested one day after transfection and solubilized with lysis buffer containing 25 mM Tris pH 7.5, 4 mM MgCl_2_, 20 mM b-glycerophosphate, Roche protease inhibitor (with EDTA) and 0.5% Triton-X 100 in PBS and sonicated for 5 seconds at 20% output. Samples were the centrifuged at 14,000 rpm for 15 minutes at 4°C. The supernatant was then passed through columns containing glutathione-sepharose beads (Amersham Biosciences). Beads were washed twice with 0.1% Triton X-100 in PBS. Bound proteins were eluted from the beads using SDS sample buffer. Samples were boiled for 10 minutes before SDS-PAGE analysis.

### Western analysis

Samples were subjected to SDS-PAGE and transferred to PVDF membranes at 100 V for 2 hours and blocked 1 hour with 10% low-fat milk. Primary antibodies were added for 2 hours at room temperature or overnight at 4°C and secondary antibodies for 1 hour. HRP-conjugated secondary antibodies were detected by Amersham ECL and Hyperfilm MP (Amersham Biosciences). All antibodies were diluted in 1% BSA in PBS.

### Wound healing assays

COS-7 or U2OS cells in 35 mm dishes were transfected with control or DAAM1 siRNA. After 24 hours the culture medium was changed to 1% FBS-containing medium and cells were incubated overnight. On the following day, a wound was made by scraping with a yellow pipette tip and cells were allowed to recover for 15 minutes and replenished with 10% FBS to drive cell migration.

### Indirect immunofluorescence and live-cell imaging

Cells were seeded on 22×22 mm coverslips in serum. Fixation was either in 3% paraformaldehyde followed by permeabilization with 0.2% Triton X-100 in PBS for 10 minutes or 100% methanol at −20°C for 10 minutes before blocking with 10% FBS for 10 minutes. Primary antibody (in PBS/0.5% Triton X-100) was added for 2 hours at 37°C and secondary antibody incubation was at room temperature for 1 hour. Images were acquired with Zeiss Axioplan 2 microscope (Carl Zeiss, Jena, Germany) using a 63x/1.4 Plan-APO-CHROMAT oil lens and a Roper Scientific CoolSNAP HQ camera or Zeiss LSM510 META confocal microscope using a 63x/1.4 Plan-APOCHROMAT oil lens. Image analyses were performed using MetaVue (Molecular Devices, Downingtown, PA) and Zeiss LSM browser (Carl Zeiss). Live-imaging of cells was performed using Olympus FluoView FV1000-MPE microscope equipped with an Olympus 60x/1.45 or 40x/1.30 Plan-APOCHROMAT lens or Olympus IX71 or Zeiss Axiovert 135 M Inverted microscope equipped with a 10x/0.25 Plan-APROCHROMAT lens. Image analyses were performed using Olympus FV10-ASW 1.6 viewer, Image-Pro Plus (Media Cybernetics, Inc, MD) or ImageJ (NIH, USA) software.

## Supporting Information

Figure S1 Interaction of DAAM1 with multiple Rho GTPases. GFP-Flag-DAAM1-N (residues 1–545) was co-expressed with GST tagged active versions of Rho proteins; GST fusion proteins were recovered on glutathione-Sepharose beads and assessed by western analysis with anti-Flag; GFP-Flag was used as a negative control.
(0.76 MB TIF)

Figure S2 U2OS cell lines stably expressing DAAM1 FL differs from control cells in terms of focal adhesion organization. Cells stably expressing HA-mCherry vector or HA-mCherry-DAAM1 FL were subjected to wound healing assays and stained with anti-vinculin to visualize the focal adhesions. Bar  = 10 µm.
(4.39 MB TIF)

Figure S3 Overexpression of DAAM1 in U2OS cells impaired migration. Cells stably expressing the HA-mCherry vector or HA-mCherry-DAAM1 FL were subjected to scratch wounding and images were acquired at 2 hour intervals over 6 hours using an Olympus IX71 microscope equipped with a 10x/0.25 Plan-APOCHROMAT lens. A representative DAAM1-expressing line (line 3) is shown. Bar  = 100 µm.
(3.26 MB TIF)

Movies S1 Migrational behavior of COS-7 cells upon DAAM1 knockdown. Control siRNA treated cells (Movies S1) or si2318 siRNA treated cells (Movie S2) were subjected to scratch wounding and analysis by phase contrast microscopy two days post-transfection. Images were acquired at 15 min intervals over 12 hours using a Zeiss Axiovert 135 M Inverted microscope equipped with a 10x/0.25 Plan-APROCHROMAT lens. Images were processed using Image-Pro Plus software. The nuclei of 6 cells were tracked in every frame using ImageJ software and the resultant paths are shown as colored lines.
(4.46 MB AVI)

Movies S2 Migrational behavior of COS-7 cells upon DAAM1 knockdown. Control siRNA treated cells (Movies S1) or si2318 siRNA treated cells (Movie S2) were subjected to scratch wounding and analysis by phase contrast microscopy two days post-transfection. Images were acquired at 15 min intervals over 12 hours using a Zeiss Axiovert 135 M Inverted microscope equipped with a 10x/0.25 Plan-APROCHROMAT lens. Images were processed using Image-Pro Plus software. The nuclei of 6 cells were tracked in every frame using ImageJ software and the resultant paths are shown as colored lines.
(3.96 MB AVI)
