# Supplementary figures and images for: DAAM1 Is a Formin Required for Centrosome Re-Orientation during Cell Migration
Source: PLoS One. 2010 Sep 29;5(9):e13064. doi: 10.1371/journal.pone.0013064 (PMC2947498; doi:10.1371/journal.pone.0013064)

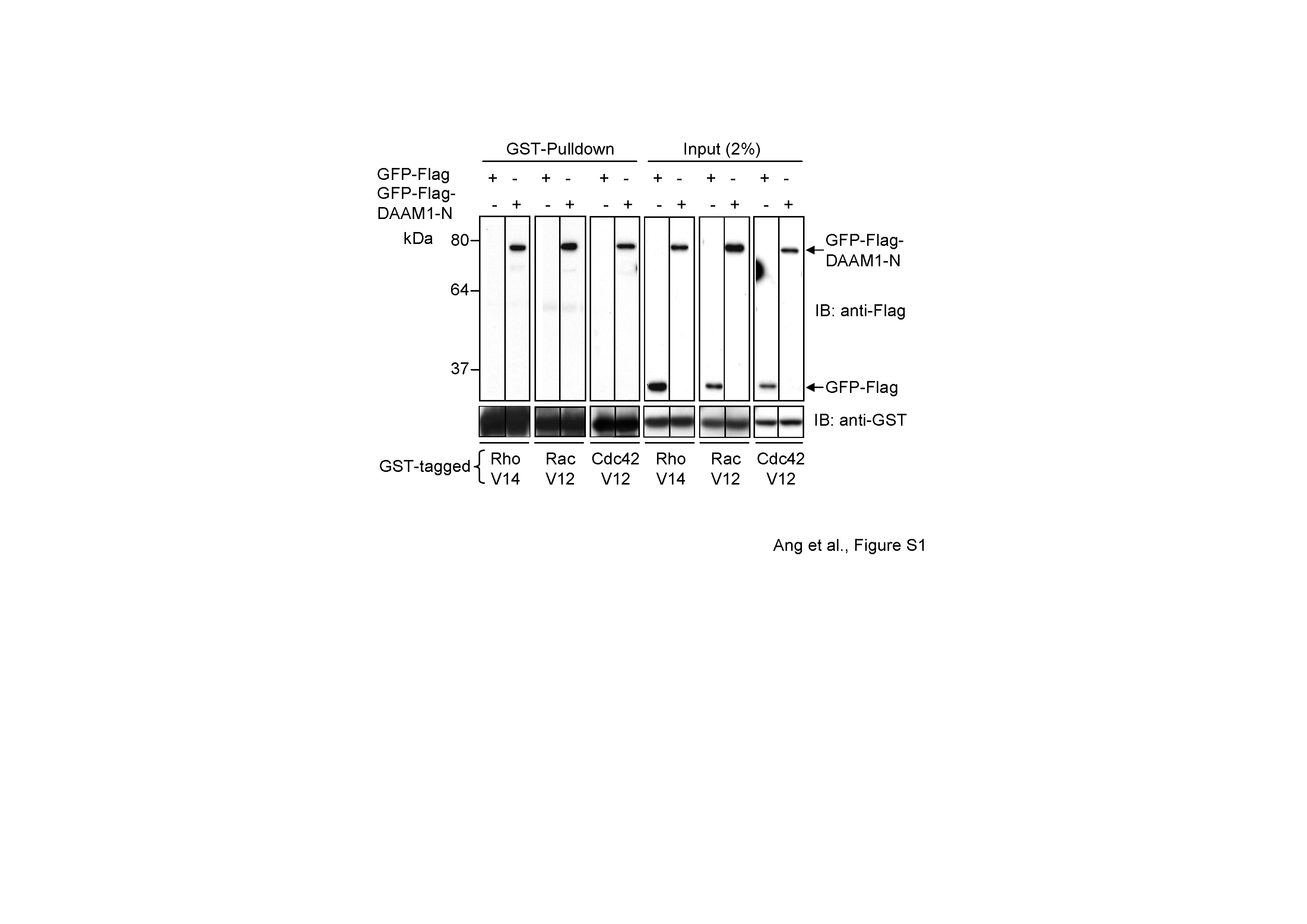

Supplement: Figure S1 — Interaction of DAAM1 with multiple Rho GTPases. GFP-Flag-DAAM1-N (residues 1–545) was co-expressed with GST tagged active versions of Rho proteins; GST fusion proteins were recovered on glutathione-Sepharose beads and assessed by western analysis with anti-Flag; GFP-Flag was used as a negative control. (0.76 MB TIF) [file pone.0013064.s001.tif]

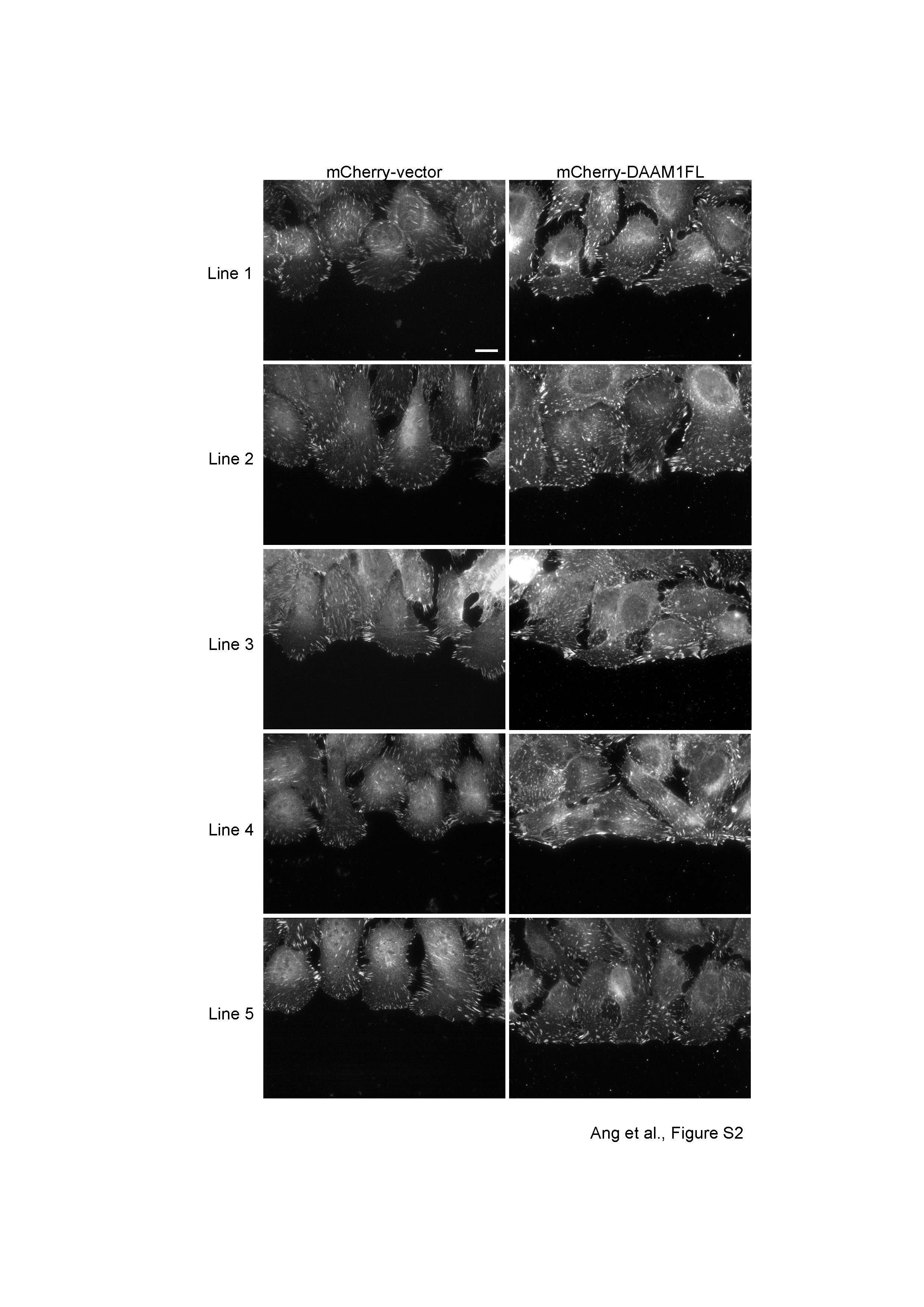

Supplement: Figure S2 — U2OS cell lines stably expressing DAAM1 FL differs from control cells in terms of focal adhesion organization. Cells stably expressing HA-mCherry vector or HA-mCherry-DAAM1 FL were subjected to wound healing assays and stained with anti-vinculin to visualize the focal adhesions. Bar = 10 µm. (4.39 MB TIF) [file pone.0013064.s002.tif]

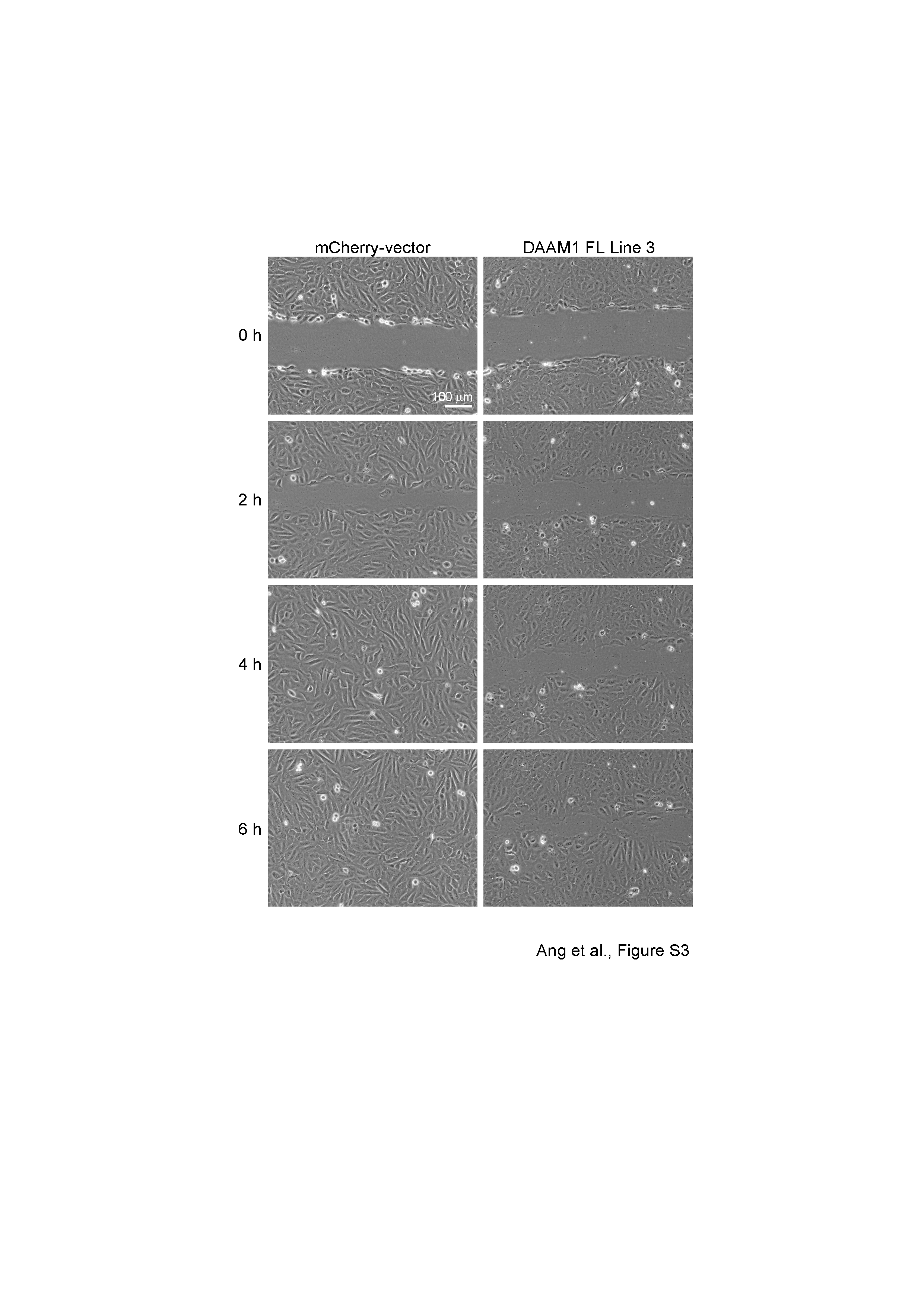

Supplement: Figure S3 — Overexpression of DAAM1 in U2OS cells impaired migration. Cells stably expressing the HA-mCherry vector or HA-mCherry-DAAM1 FL were subjected to scratch wounding and images were acquired at 2 hour intervals over 6 hours using an Olympus IX71 microscope equipped with a 10x/0.25 Plan-APOCHROMAT lens. A representative DAAM1-expressing line (line 3) is shown. Bar = 100 µm. (3.26 MB TIF) [file pone.0013064.s003.tif]
